# Development and validation of a five-level developmental model for new graduate employees

**DOI:** 10.1007/s43545-022-00420-w

**Published:** 2022-08-02

**Authors:** Yoshiko Goda, Kentaro Sudo

**Affiliations:** 1 Research Center for Instructional Systems, Kumamoto University, Kumamoto, Japan; 2 Research and Development Dept., Alue Co., Ltd., Tokyo, Japan

**Keywords:** New employee, Graduate hiring, Development model, Criteria for performance

## Abstract

The aim of this research was to create a developmental model for new graduate employees according to improvements in their performance. The model’s scope covered the period when employees take part in apprenticeship training after graduation from college or university. The process of developing and validating the model referred to the International Board of Standards for Training, Performance, and Instruction’s competency development model as a framework and involved six steps. This research analyzed 111 freshly graduated recruits who had recently been hired by a leading travel company in Japan. The final model includes five apprenticeship levels, namely beginning, elementary, intermediate, advanced, and end of apprenticeship, with criteria for performance in planning and implementation. To validate the model, three transitions in the developmental levels over six months in 2017 and nine months in 2020 were examined using the model’s performance criteria. The results show that the transitions for the three companies reflect the characteristics of their new graduate employees. This indicates that the proposed model may be valid for determining the development levels of new graduate recruits and can have a certain level of discriminative power. This model can serve as a helpful tool for planning on-the-job and off-the-job training to support new employee development, comprehending their current development levels, and providing a guideline for the next step for further development.

## Introduction

The importance of training and supporting new employees has been emphasized for some time. Louis et al. (1983) reported that 64% of organizations provided new employee orientation programs, a figure reported to be 70% by Zenke (1982). Some studies have discussed the content and instructional design of such programs (e.g., Acevedo and Yancey 2011; Wanous and Reichers 2000). Despite this long history of research, little of it has been conducted on new employee development with a focus on newly hired graduates and their first experiences of the workplace after entering the workforce. Many studies have focused on how new employees (newcomers) are onboarded at target companies, albeit on recruits with a variety of previous work experience (e.g., Bauter et al. 2007; Korte and Lin 2013; Newstrom 1983). However, studies with an emphasis on newcomers without real fulltime work experience and hired straight after graduating from university are limited. One of the main reasons for this is that it is difficult to obtain data by observing a substantial number of new graduate employees to investigate their developmental paths.

As Mestre et al. (1997) explained, the Japanese situation is unique, and most large companies still hire a considerable number of fresh graduates each April, at the start of the new financial year. According to a survey of the employment status of university graduates in 2018 by MEXT (2019), the employment rate of undergraduates was 97.6% in Japan. Due to such a high employment rate, Japanese companies constitute an optimal research setting for examining how recently graduated employees develop social and business skills and knowledge as well as ways they grow as members of society. Moreover, in light of the uniqueness of Japan’s prevailing system for employing fresh graduates, the steps taken by new employees in advancing in their career development toward becoming business professionals can be extensively researched.

The aim of this research is to develop a model of professional development for fresh graduates according to the evolution of their work performance. The model’s scope covers the period during which employees receive apprenticeship training after their graduation from college or university. This model will serve as a powerful tool for planning on-the-job (OJT) and off-the-job (Off-JT) training in support of new employee development.

## Background and related literature

### New employee development

New employee development is defined as “all development processes used to advance new employees to desired levels of performance” (Holton 1996, p. 233). Holton described two expected outputs of new employee development as their attainment of a targeted level of performance and staying with an organization. To support a new employee in reaching a specific performance level, that level should be defined. The departure rate of fresh graduate recruits within 3 years may be as high as 31.8% in Japan (MHLW 2018). Appropriate orientation and support may reduce this rate among new employees.

Holton (1996) proposed the New Employee Learning Taxonomy, which was based on Fisher’s four content domains for learning among new employees—individual, people, organization, and work tasks—for designing learning content and strategies. The taxonomy could be helpful for designing tasks for training programs and learning environments; however, it does not include development paths or consider levels of performance development and how new employees can improve their performance. Thus, the taxonomy cannot be used to assess new employees’ developmental levels. It is necessary to assess such levels and steps of individual development to examine more effective ways to facilitate their professional growth.

Holton and Naquin (2004) reviewed and critiqued existing metrics for employee development. He reviewed six different measuring techniques, namely the financial, intellectual capital, HRD evaluation, American Society for Training and Development, human resource/performance research, and human resource metrics approaches. Nevertheless, these are not enough to fully capture the development of employees. Holton also proposed development metrics created at three levels: organization or organization sub-unit level, intervention or program level, and individual employee level. The items of his metrics include development quality, capacity to meet potential needs, development of customer satisfaction, formal development investment per employee, and human capital development contribution. All four items include formulas to calculate values and collectively evaluate employees’ development for determining their impact on a business. However, the metrics focus on all employees, and not only new ones. In this article, a developmental model is proposed for individual fresh graduate recruits, rather than one for their organizational impact. In this research, the model’s scope covers the period of apprenticeship training for recruits following their graduation from college or university. Manualized and patterned types of work are used for the model.

### Onboarding of new employees

Research using the term “onboarding” first appeared in the literature around the early 2000s. Bauer (2010) defined onboarding as “the process of helping new hires adjust to social and performance aspects of their new job quickly and smoothly (p. 1).” As the popularity of onboarding research has increased, scholars have identified the problem of retaining newcomers. Ellis et al. (2017) reported that about 17% of newcomers depart during their first three months of employment, and Bauer (2010) indicated that half of senior outside hires leave within 18 months and half of hourly workers quit within four months. The research on employee turnover and reasons for leaving suggests the need for companies to focus on organizational commitment, better job satisfaction, less work stress (Sree Rekha and Kamalanabban 2010), and employee training (Govaerts et al. 2011) to improve employee retention. These findings are not limited to newcomers, as socialization and learning opportunities are key for all employees to reduce their intention to leave.

One of the most cited socialization models is that of Van Maanen and Schein (1979). The model includes six tactical dimensions for socialization processes: collective versus individual, formal versus informal, sequential versus random steps, fixed versus variable, serial versus disjunctive, and investiture versus divestiture. Individual newcomers’ onboarding and socialization are affected by previous experiences (Korte and Lin 2013). Two major problems have arisen in previous research on the onboarding of new employees: one is the lack of a learning perspective (Klein and Heuser 2008) and the other concerns dealing with newcomers as a homogeneous group and “blank slate” (Becker and Bish 2019). Becker and Bish (2019) pointed out that the perspective of what newcomers require should be considered instead of what information organizations need to impart. Socialization is an important key for onboarding. Becker and Bish (2019) emphasized the importance of unlearning previous experience for smooth onboarding. In this research, blank slate newcomers are targeted, and their learning processes and daily challenges are closely observed and analyzed.

### Apprenticeship

There is not one universal definition for apprenticeship, and its meaning varies across countries in terms of quality (skill content) and quantity (numbers trained) (Wolter and Ryan 2011). Moon (2018) took definitions from two dimensions: (a) a process-based perspectives including learning styles (Nonaka et al. 2000), processes (Kang 1996), and outcomes (Brown et al. 1989) and (b) an operational perspectives including long-term operations (Lee et al. 2014) and internships (Yoo 2012). Ericsson (1990) proposed a 10-year rule with deliberate practice to become an expert in almost anything. In expertise research, several hierarchical and developmental models have been introduced. Dreyfus and Dreyfus (1986) updated their five-stage model of skill acquisition with a seven-stage model: novice, advanced beginner, competence, proficiency, expertise, mastery, and practical wisdom. The novice stage is the period during which a person with no work experience becomes aware of and understands the situations around them. A four-stage model constructed by Kanai and Kusumi (2012) was employed to determine the span of individual development in this research, namely beginner, competent, middle-ranking, and expert. Here, the beginner stage is described as lasting 1 year, and then 3 to 4 years are usually required to reach the next stage, competent. The scope of the present study overlaps with the beginner and competent stages. The former includes initiation and adapting to work and colleagues, learning and observing under the guidance of a trainer or supervisor, and mastering general procedures, skills, and rules through apprenticeship. In the beginning, the employee makes more mistakes, but the mistakes gradually become fewer. The latter stage is characterized as gaining routine expertise. In this research, the focus is on new graduate employees, and the period considered in the proposed model is for 1 to 4 years during the beginner and competent stages mentioned above.

Moon (2018) reviewed literature related to apprenticeship and developed a conceptual model to connect the stages from newcomer to professional via onboarding and the developmental and proficient phases. Since this research focuses on new recruits’ initiation in the workplace, its span is matched with Moon’s onboarding phase, which has three components (inform, welcome, and guide), leading to socialization with individuals. The inform component includes communication, resources, and training, while the welcome component consists of celebrating, expressing appreciation, and providing opportunity to meet other organizational members. Finally, the guide component has two elements: providing active, direct assistance and differentially onboarding different types of employees. This model is useful for designing activities and resources for supporting a newcomer in becoming part of an organization, but it does not provide information on the process of new employees actually developing skills, knowledge, and attitude for work. This study aims to provide the steps and levels of development for new employees during the first stage of their apprenticeship.

### Human resources in Japan

According to OECD (2022a), Japan’s unemployment rate in 2020 was the lowest (2.77%) in the organization, compared to Costa Rica, with the highest unemployment rate (19.61%), and Greece (16.44%). The US’s unemployment rate was 8.09%, and that of the 19 countries of the Eurozone was 7.94%. Countries with a relatively low unemployment rate included Germany, the Czech Republic, and Israel. The OECD’s unemployment rate by age group (15–24) also showed Japan to have the lowest rate, at 5.2% (OECD 2022b). This low rate of unemployment among young people in Japan is reflected by the hiring system for new graduates in the country (MHLW 2013). The cases of other countries are taking some approaches to lower unemployment, for example, Germany uses the renewed training and institutional reform (Lorents 2016).

Japan’s culture is seen to have a higher power distance, whereby those in authority have the most power. There are also high levels of collectivism and less individualism, with people placing greater emphasis on harmony (Hofstede 1983). In comparing Japanese culture with American culture, the Hofstede cultural index for power distance gave Japan a score of 54 and a rank of 21 out of 50 countries across three regions. In comparison, the US scored 40 with a rank of 16. The index for individualism–collectivism gave the US a score of 91 and ranked it in 50th place, compared to Japan’s score of 46 and ranking of 28–29. The ranking was ordered from the highest level of collectivism to the lowest, which in turn indicated the most individualism. However, in recent years, research has reported that cultural characteristics have changed since the time of Hofstede’s original research.

Although Japan maintains a low unemployment rate compared to other countries, the departure rate for new employees within 3 years of starting work is as high as 36.9% for high school graduates and 31.2% for those with an undergraduate degree (MHLW 2018). Among the reasons for Japan’s high departure rate of new employees is the mismatch in the job market. The hiring system for fresh graduates has issues, such as students’ preference for working at larger and better known companies. Since large companies and small and medium-sized enterprises recruit students as employees at the same time, it is critical that they attract the best possible candidates. Potential employees who have gained information through internships and corporate briefings by skilled human resources departments tend to apply for positions at large, well-known companies. Small and medium-sized enterprises, however, are prevented from doing this because of cost and personnel issues. Another reason for Japan’s high departure rate is that new employees feel unable to develop their skills. Working environment and welfare are also factors that influence newcomers’ departure from a company.

To reduce the departure rate of the new employees, onboarding, mentoring, Off-JT, and OJT are now common in Japan, and they are seen to be effective in helping newcomers to adapt quickly to their new environment and feel useful as contributors to the company at an early stage. Currently, training and support for new employees are emphasized straight after they start work at a company, and about 70% of employees are provided with Off-JT by their employer (MHLW 2014). The cost for training one employee was about ¥35,000 in 2019, although it decreased to about ¥25,000 in 2020 (Sanro Resaerch Institute 2021).

In Japan, companies’ human resource development involves different levels of education and training, such as OJT and Off-JT, and reassignment. In recent years, companies have recognized that a lack of guidance and training for staff and overwork among bosses are major issues facing human resource development managers in Japan (MHLW 2013).

## Research purpose and questions

Since the required skills, knowledge, and attitude for each developmental stage are unknown, a planned career development model for new graduate employees would be helpful for designing and implementing onboarding and OJT/Off-JT for new employees. The objective of this research is to (a) propose a valid and reliable development model for new employees for when they start work after graduation and (b) provide criteria to define each level of the model so that it can be used to design and help new employees’ development. Proper design and implementation of onboarding and OJT/Off-JT, along with self-development at the early stage of their career, should provide new employees with smooth and successful career development. In this study, there are two research questions relating to these goals:

1. How do new employees develop their skills, knowledge, and attitude in a company?
2. What criteria can be used to define the development levels?

## Methodology

### Research field

This research targeted one of the leading travel agencies in Japan (Company A), which employed 2247 staff as of April 1, 2019. With four branches in Tokyo and 14 elsewhere in Japan, each year the company hires some 120 college graduates. In 2017, 113 graduate employees (male: 49 [43%], female: 64 [57%]) were hired. To validate the preliminary model for this research, the data of 111 graduate employees (two staff resigned prior to the study) were taken from 2017 data. The company has improved its new employee development program, and the support it provides to better develop new employees led to a reduction of its departure rate within 3 years. Company A usually provides newcomers with face-to-face training in April and May and OJT in their assigned department at the beginning in June. New employees are required to submit a monthly progress report to the company. During OJT, a new employee is directly guided by two senior staff members, while OJT leaders supervise the senior staff. The company agreed with the research motivation, whereby new graduate development levels could contribute to a more effective and efficient implementation of developing the skills of recruits, and therefore was willing to participate in this research study.

### Ethical considerations for research participants

The participants were part of the study by default, since the company had chosen to participate in it. A consulting company that provided training and development for employees signed a MOU with Company A relating to data collection and analysis that would better support the employees’ development. The data used in this research were collected through the daily reports of trainers. These data were not provided specifically for research purposes, but the trainers’ reports for a whole year were utilized by this research. During the data analysis, anonymity was maintained, and the participants are not identifiable to others, including their supervisors. The risks to human subjects were minimal, and no serious concerns were found.

### Scope of the model’s development

The purpose of model development is to clarify the developmental levels of fresh graduate recruits’ performance. The model might be utilized by HR professionals for OJT and Off-JT training, environment, and learning support and by new employees to establish performance goals with which they can cultivate their own skills and knowledge. The scope of the developmental model covers the growth of new graduate employees during their early period in a new job, probably in the first and/or second year of their careers.

### Steps to develop and validate a development model for hiring new graduate employees

**Fig. 1:**
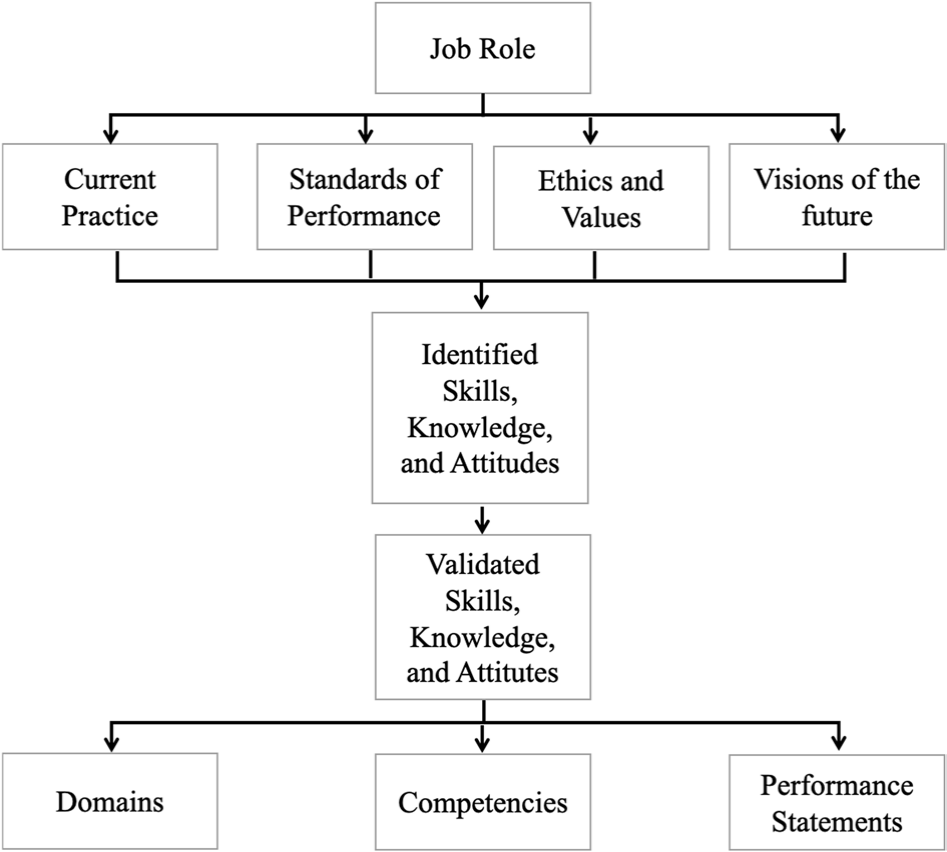
IBSTPI competency development model (Klein and Richey 2005, p.10)

For the procedures of model development, the International Board of Standards for Training, Performance, and Instruction (IBSTPI) competency development model was employed as a framework (Klein and Richey 2005, p.10; Fig. 1). This model is used to develop international standardized competencies for professionals and includes several steps to identify required knowledge, skills, and attitudes along with job tasks and capabilities for the targeted occupation, profession, or organizational role. The rationale for using the framework is that the developmental model in this research has levels, and each level has targeted behavior and competencies, so a framework to identify the competencies would be a good guide. This is because the research process and views and perspectives for validation of the competencies are helpful for examining the proposed model with the competencies at each level. The research outcomes of this research match to the IBSTPI model; (a) descriptions of each level in the proposed model for new employees as the competencies in the IBSTPI model, and (2) the proposed criteria in this study as the performance statements in the IBSTPI model.

After consulting the IBSTPI model, the process of developing and validating the model involved six steps: (a) development of a preliminary model, (b) validation based on theories and previous literature, (c) expert review of the preliminary model, (d) continuous checks to analyze the reports, (e) subsequent expert review of data analyses from the model, and (f) evaluations both by new employees and their supervisors. Table 1 summarizes the steps and provides an overview of the validation, including the dates. The validation process is discussed as follows, according to each step.

**Table 1:** Steps to develop a new employee model

Step	Action	Date	Description
1	Development of a preliminary model	Dec. 2018	A preliminary model was made for new employee performance development It involved a review of existing literature and practices (current practices and standards of performance)
2	Validation (1) Theories	Jan. 23, 2019	The model was revised using Bloom’s learning taxonomy, especially in the areas of cognitive, affective, and self-regulated learning After the revision, the behavioral categories of Bloom’s taxonomy were applied to revisit the expressions and definitions of each level of the model
3	Validation (2) Expert review (1)	Feb. 2019	This expert review showed positive agreement between the various levels of the model. The differences between levels 3, 4, and 5 were modified based on the results of the interviews
4	Validation (3) Data analysis (1)	May 2019	Two evaluators analyzed the monthly reflective reports (September 2017 to January 2018) of 20 new employees; however, due to inconsistent results between the beginning three levels, the model was revised to include six levels by the end of this step
5	Validation (4) Expert review (2)	May 13 and 24, 2019	Under the HR expert’s observation, the six-level development model was viewed as predominantly consistent
6	Validation (5) Data analysis (2)	Jun. 27, Jul. 22, Jul. 25, Aug. 19, and Aug. 29, 2019	To validate the model, the monthly reflective reports were analyzed three times by two evaluators. First, the data of 14 new employees were analyzed and appropriated to one of the different levels of the model. The evaluators discussed the inconsistent results and their degree of agreement, and the expressions and definitions of each level were then revised. Second, the evaluators categorized an additional 26 employees’ reports into one of the model’s levels; the same procedures for inconsistent results were implemented, and the model was subsequently revised. Third, an additional 30 employees’ reports were analyzed to confirm the model’s accuracy. Following this, a new evaluator categorized these employees according to their levels of progress and referred to the model to ensure that it could be used as a criterion for the correct appropriation of their development. Lastly, the original two evaluators rated the at-the-point level for all 111 new employees based on their individual monthly reports

#### Step 1: development of a preliminary model

A preliminary model for new graduate employee development was proposed by two business training professionals (see Table 2). As consultants, they had been responsible for employee development at one Japan’s top business training companies for more than 10 years. This step involved a review of the existing literature and practices (current practices and standards of performance). The practices included expert observations, employees’ interactions with stakeholders, including with human resources professionals, trainers, supervisors, and other employees at different companies, and their experiences from their own OJT training.

**Table 2:** Preliminary model of development

Level	Apprenticeship level	Work type	Description of recruit’s level of development
1	Beginning of apprenticeship		S/he learns the basic procedures and methods of the work and completes tasks accordingly
2	Intermediate apprenticeship	Manualized work	S/he handles more than one type of task and has learned basic procedures and methods S/he can finish tasks that have previously been worked on, before or on schedule S/he is aware that s/he does not know about work procedures
3	Advanced apprenticeship	Manualized work	When being given a job or getting started on it (e.g., making a business plan), s/he reviews unclear points and set-up instructions
4	End of apprenticeship	Patterned work	In consideration of post-process tasks, s/he may start projects that require further consideration after handing work over to others (includes reviews)
5	Beginning of independence	Non-patterned work	S/he can achieve the intended results more often than expected S/he improves routine work and adds further ingenuity to work that has been delegated to her/him

Although there were five levels of development, the process ended at Level 4, with Level 5 being the start of the recruits’ independence. The preliminary model was modified and revised based on the following validation steps.

#### Step 2: validation (1) theories

The model was revised using psychological and educational theories and models from the perspective of cognitive and affective domains (see Table 3). The order of the developmental levels was examined with a reliable framework and theories for this step.

**Table 3:** Second step of validation: referenced theories and models

Research interest (RI)	Level	Apprenticeship level	Work type	Cognitive domain		Affective domain		
				Cognitive domain in Bloom’s taxonomy	Self-regulated learning development (Schunk and Zimmerman 1997)	Affective domain in Bloom's taxonomy	Four-Phases of interest development (Hidi and Renninger 2006)	Self-determination theory of motivation (Dici et al. 1996)
Out of RI	6	Beginning of independence	Non-patterned work	Evaluation	Level 4: Self-regulation	Valuing	Phase 4:Well-developed individual interest	Internal motivation
Within RI	5	End of apprenticeship		Synthesis	Level 3: Self-control		Phase 3: Emerging individual interest	Integrated regulation
	4	Advanced apprenticeship	Patterned work	Analysis		Responding		Identified regulation
	3	Intermediate apprenticeship		Application	Level 2: Emulation			
	2	Elementary apprenticeship	Manualized work	Comprehension			Phase 2: Maintained situational interest	Introjected regulation
	1	Beginning of apprenticeship		Knowledge	Level 1: Observation	Receiving	Phase 1: Triggered situational interest	External regulation

The cognitive domain in Bloom’s learning taxonomy and self-regulated learning development (Schunk and Zimmerman 1997) were applied to assess the model based on the cognitive domains. The taxonomy and theory were selected because they had well-established validity, were popularly applied to self-learning, and showed a definite hierarchy from the lower to higher or from novice to advanced development. For the affective domain of the proposed model, the affective domain of Bloom’s learning taxonomy, the four phases of interest development (Hidi and Renninger 2006), and the self-determination theory of motivation (Dici et al. 1996) were applied.

For example, to develop self-regulatory competence, trainers would guide their trainees through four levels of self-regulated learning (SRL) development, namely observation, emulation, self-control, and self-regulation (Reynolds et al. 2003; Schunk and Zimmerman 1997; Zimmerman 2000, 2013). The hierarchical orders of the model were reviewed and examined to ensure that they were consistent with SRL development. Based on the theories and models of the previous research, the hierarchical orders of development in this model and the expressions and definitions of each level were carefully revised and modified.

#### Step 3: validation (2) expert review (1)

An expert participated in the first expert review. She had worked in an HR department for more than 9 years and observed new graduate development. This expert acknowledged a positive agreement between the various levels of the model. She pointed out that the performance statements of Levels 3, 4, and 5 were unclear and overlapped each other. Thus, the differences between Levels 3, 4, and 5 were modified based on the results of the interview.

#### Step 4: validation (3) data analysis (1)

Two evaluators analyzed the monthly progress reports (September 2017 to January 2018) of 20 new employees. Due to inconsistent results between the beginning three levels, the model was revised to include six levels by the end of this step (see Table 4). To clarify the levels between the end of apprenticeship and beginning of independence, the model had six levels; however, Level 6 was out of the research scope within the apprenticeship, and thus it was removed from the proposed model.

**Table 4:** Modified six-level model, including beginning of independence

Level	Apprentice level	Work type	Description of recruit’s level of development
1	Beginning apprenticeship		When trying to understand and implement instructions for routine work, the work may not go as planned or follow the established procedure S/he only partially understands the given instructions and relies on her/his own interpretation to follow procedure There are scenarios in which errors in recognition lead to mistakes and delays
2	Elementary apprenticeship		S/he understands the instructions for routine work and works according to determined procedure Although s/he is aware of deadlines and attempts not to inconvenience other staff, s/he may delay her/his reports to senior staff members until right before a task is due
3	Intermediate apprenticeship	Manualized work	S/he grasps tips and advice for skillfully carrying out routine work, proceeds with a certain expected business outlook, and is willing to take on challenging new tasks on his/her own S/he still requires guidance and support from leaders during certain tasks. However, s/he is capable of self-management and reports and communicates in a timely manner when her/his work is disrupted
4	Advanced apprenticeship	Manualized work	Routine work is carried out competently on her/his own, and other work is completed in cooperation with coworkers, taking into account how this work may impact priority tasks To expand the scope of her/his capacity, s/he is willing to take on new work, increase work knowledge, and imitate the ways that colleagues and supervisors complete tasks
5	End of apprenticeship	Patterned work	S/he is aware of the goals and objectives of the team and follows up and supports the work of both the person in charge and her/his coworkers If her/his team members have the necessary information, s/he is willing to share or act in a contributing manner S/he is aware of the team’s results and aims to independently raise the level of the results of both priority and non-priority tasks
6	Beginning of independence	Non-patterned work	S/he understands the level of achievements and results that are expected from the organization and carries out operations that meet these standards S/he aims for better results and products by making improvements, such as proposals to enhance existing operations, referring her/his criteria to the results and products submitted to end-customers

#### Step 5: validation (4) expert review (2)

Two HR experts participated in the second expert review. The five-level model developed as a result of validation step 4 was reviewed by them. One of the experts was also part of the first expert review, and the other expert had more than 5 years’ HR experience. Under the HR experts’ observations, the development model was viewed to be predominantly consistent.

#### Step 6: validation (5) data analyses (2)

To validate the model, the monthly progress reports (September 2017 to February 2018) were analyzed three times by two evaluators and once by a new evaluator (see Step 6 in Table 1). First, the six monthly reports of 14 new employees were analyzed and appropriated to one of the different levels of the model. The initial interrater correlation was 78.6%. The evaluators discussed the inconsistent results and their degrees of agreement, and the expressions and definitions of each level were then revised. Second, the evaluators categorized an additional 26 employees’ reports into one of the model’s levels, following the same procedures for inconsistent results, and the model was subsequently revised. Third, an additional 28 new employees’ reports were analyzed to confirm the model’s accuracy. Following this, a new evaluator categorized the employees according to their respective level of progress, referring to the model to ensure that it could be used as criteria for the correct appropriation of their development. Lastly, the original two evaluators rated the at-the-point level for all 111 new employees separately, based on their monthly reports. The interrater correlation was 77.2%, but about 90% of the inconsistent results were caused by carelessness of either evaluator; thus, the final interrater correlation reached 97.7%.

## Results

**Fig. 2:**
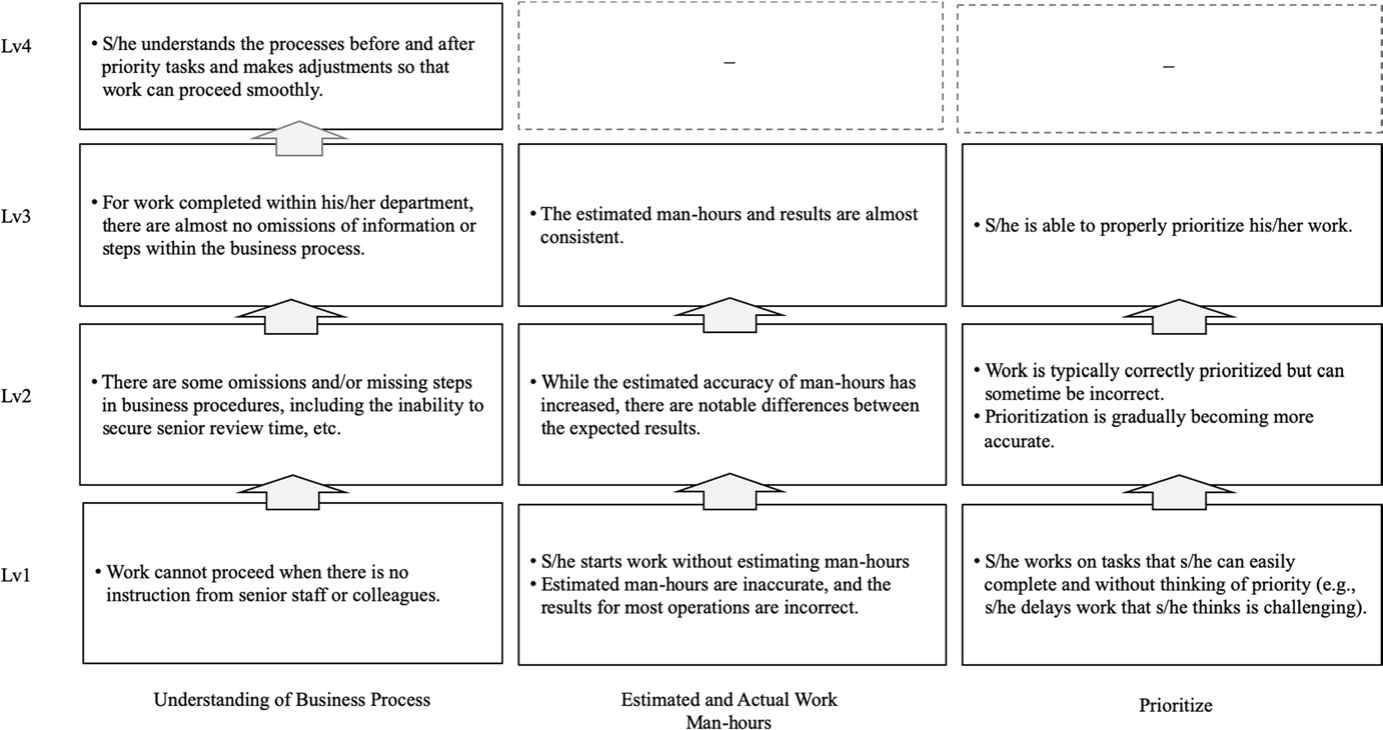
Levels of criteria for the planning phase

**Fig. 3:**
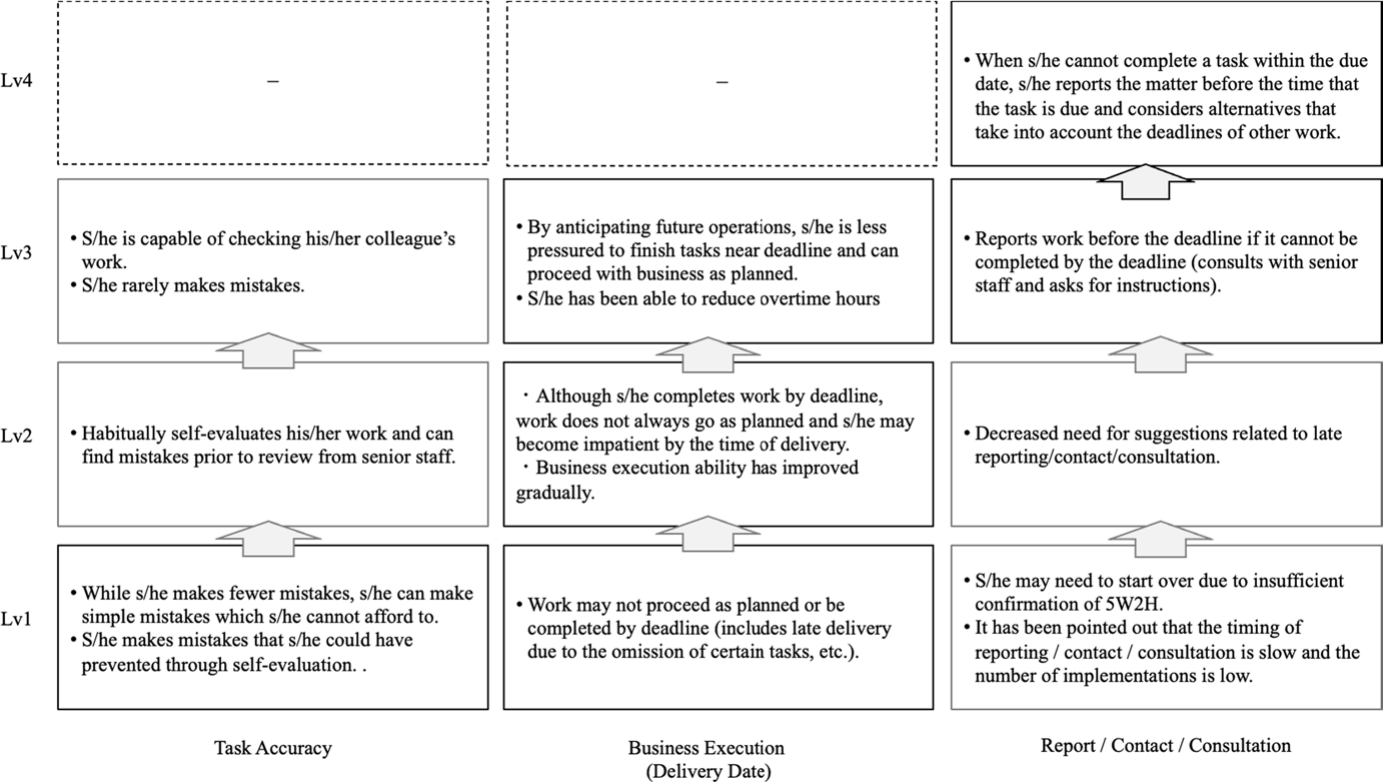
Levels of criteria for the implementation phase

The final model includes five apprenticeship levels, namely beginning, elementary, intermediate, advanced, and end of apprenticeship (see Table 5), with criteria for performance in planning (Fig. 2) and implementation (Fig. 3).

**Table 5:** Final version of the five-level model of development for new graduate employees

Level	Apprenticeship level	Work type	Description of recruit’s level of development
1	Beginning apprenticeship		When trying to understand and implement instructions for routine work, the work may not go as planned or follow the established procedure S/he only partially understands the given instructions and relies on her/his own interpretation to follow a procedure There are scenarios in which errors in recognition lead to mistakes and delays
2	Elementary apprenticeship		S/he understands the instructions for routine work and works according to the determined procedure Although s/he is aware of deadlines and attempts not to inconvenience other staff, s/he may delay her/his reports to senior staff members until right before a task is due
3	Intermediate apprenticeship	Manualized work	S/he grasps tips and advice for skillfully carrying out routine work, proceeds with a certain expected business outlook, and is willing to take on challenging new tasks on her/his own S/he still requires guidance and support from supervisors during certain tasks However, s/he is capable of self-management and reports and communicates in a timely manner when her/his work is disrupted
4	Advanced apprenticeship	Manualized work	Routine work is carried out competently on her/his own, and other work is completed in cooperation with coworkers, taking into account how that work may impact priority tasks To expand the scope of her/his capacity, s/he is willing to take on new work, increase work knowledge, and imitate the ways in which her/his colleagues and supervisors complete tasks
5	End of apprenticeship	Patterned work	S/he is aware of the goals and objectives of the team and follows up and supports the work of both the person in charge and her/his coworkers If her/his team members have the necessary information, s/he is willing to share or act in a contributing manner S/he is aware of the team’s results and aims to independently raise the level of the results of both priority and non-priority tasks

### Final version of the five-level model of development for new graduate employees

Table 5 presents the final model of the five levels of new graduate employee development. Level 1 (beginning of apprenticeship) up to Level 3 (intermediate apprenticeship) deal with the manualized work type.

New employees in Level 1 are at entry-level apprenticeship. They tend to understand part of instructions for routine work, and their lack of the comprehension of the work leads to mistakes and delays.

A new employee at this level has difficulty planning and does not check relative work other than the part of the job assigned to him or her for immediate attention. Level 2 employees are at an elementary of apprenticeship, and their comprehension of routine work instructions improves concerning deadlines. Despite their awareness of work deadlines, such employees tend to have problems with their estimations of these due to their naive metacognition and likelihood of delaying taking advice from senior staff members until right before the task is due. At Level 2, an employee is expected to perform properly according to their instructions. Level 3 employees enter into intermediate apprenticeship. They have a faster comprehension of their instructions and skillfully carry out routine work while maintaining an expected business outlook. They start showing interest and will continue to seek guidance and support from managers for challenging new tasks. They demonstrate a certain level of just-in-time reporting for disrupted work. At this level, employees begin to demonstrate self-management and skills regulation, and mistakes and problems related to work decrease for manualized work.

At Levels 4 and 5, the work type changes from manualized work to patterned work. Level 4 advanced apprenticeship employees carry out routine work at a professionally satisfactory level by themselves. Work beyond routine tasks is completed in cooperation with coworkers and in consideration of the features, significance, and impacts of the work. They also become willing to take on new work, show an increase in their working knowledge and engage in meaningful interactions with colleagues and managers to complete tasks. At Level 5, end of apprenticeship employees show signs of independence from the apprenticeship and of proper socialization. They are aware of the goals and objectives of the team and connections between their work and that of others. They become willing to share what they know with others and contribute to the society they belong to. They consider results as a team and work independently to raise the level of the results of both priority and non-priority tasks. The higher the level that these employees reach, the more self-regulation, self-management, and socialization they demonstrate.

### Performance criteria

In addition to the five-level model, two criteria were developed to assist researchers, practitioners, and new employees to utilize the model properly, and two criteria were also developed to focus on the performance of new employees at planning (see Fig. 2) and implementation (see Fig. 3) phases.

#### Levels of criteria for the planning phase

The criteria for the planning phase involve three viewpoints: business process understanding, estimated and actual man-hours, and prioritization. During manualized work up to Level 3, new employees are expected to become familiar with and accurately estimate the workload and prioritization of the work process.

#### Levels of criteria for the doing phase

The criteria levels are categorized from the perspective of task accuracy, including mistakes, business execution (delivery date), and report/contact/consultation. Business execution and communication involving reporting/contacting/consultation are basic business skills. At the end of the manualized work, employees are expected to master these basic skills. After mastering the basic skills in the planning and doing phases, the employees are also expected to reduce mistakes in the manualized work up to Level 3.

## Validation of the model: comparisons among three companies

To validate the model, new graduate employees’ transitions to achieve each level were organized and compared across three companies to examine the model’s discriminative power. First, the model was applied to Company A, whose data were used to develop the model. It was then applied to two other companies (Companies B and C), each with a different work focus, culture, and mission, to assess whether the model could capture the differences between them.

### Transition of achieved levels from September 2018 to February 2019

**Fig. 4:**
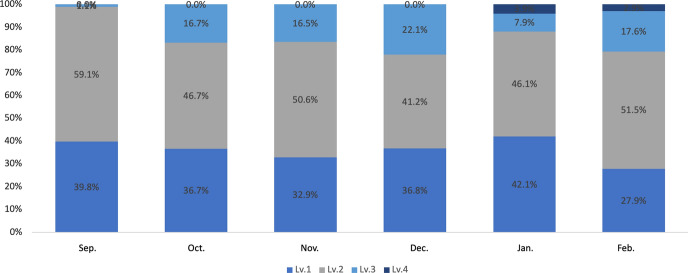
Transition of Development Levels for New Employees in Company A between September 2018 and February 2019

Transition through the developmental levels of the targeted employees of Company A between September 2018 and February 2019 was examined using the model’s performance criteria for 111 new graduate employees. The results of the transitions are shown in Fig. 4. The results demonstrate the percentage change of each level, from 39.8 to 27.9% at Level 1, 59.1 to 51.5% at Level 2, 0% to 17.6% at Level 3, and 0% to 2.9% at Level 4. There were no Level 5 employees by the end of the first working year.

### Transition of achieved levels from June 2020 to March 2021 of companies B and C

**Fig. 5:**
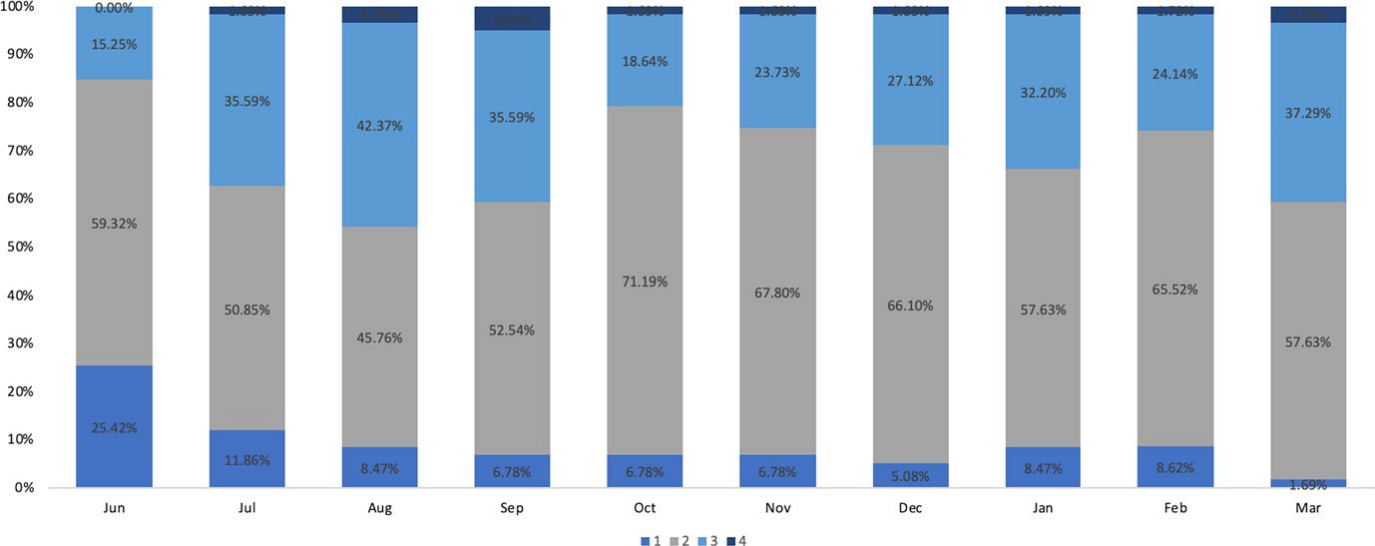
Transition of development levels for new employees in company B between June 2020 and March 2021

**Fig. 6:**
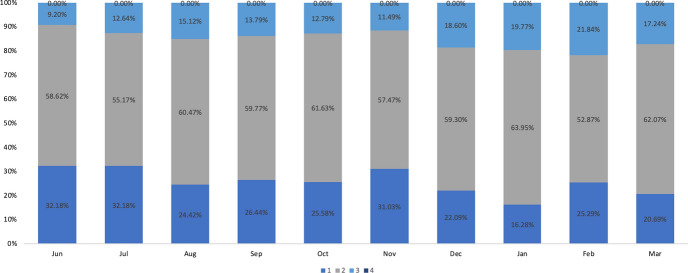
Transition of development levels for new employees in company C between June 2019 and March 2020

The transitional levels for the development of new graduate recruits at Companies B and C are shown in Figs. 5 and 6, respectively. Company B is a medical company, and new employees are expected to become medical representatives. Company C is a construction and engineering company. Data were collected for 60 new employees in Company C.

One of the key factors affecting newcomers’ socialization in a company is its culture. Cameron and Quinn (2011) introduced four types of organizational culture: adhocracy, clan, hierarchy, and market. They used two dipole dimensions for the four types of culture: flexible and stable structure and inward and outward focus. Adhocracy is a type with a flexible structure and outward focus whose characters are dynamic, entrepreneurial, and innovative. Clan, a flexible structure and inward focus culture, is characterized as having value cohesion, participation, and resembling a personal place, like a family. Market has a stable structure with an outward focus and is characterized as being results-oriented, willing to get the job done, and valuing competition and achievement. A hierarchy has a stable structure and an inward focus that is characterized as favoring structure and control, coordination, efficiency, and the importance of stability. The data used for the model development were retrieved from the new employees of Company A, a company with a clan culture.

First, the organizational culture of each company used for the model validation was determined through information collected from the company’s HR officers and a third-party consultant with about 15 years of experience in the profession. Company B’s organizational culture was categorized as a market, and that of Company C as a hierarchy. The criteria for selecting new employees were originally different, and the organizational cultures were also different among the three companies utilized for the validation of the model. To validate the usefulness of the model, the transitions of the achieved levels of the new recruits in the three companies were compared. If the transitions demonstrate differences among the companies, the model and criteria for the levels should have a certain level of discriminable power.

The transitional levels of the development for Companies B and C’s new graduate recruits are organized in Figs. 5 and 6. When the levels achieved in March 2021 are compared between the two companies, Company B consisted of 1.69% of employees at Level 1, 58.63% at Level 2, 37.29% at Level 3, and 3.39% at Level 4. About 40% of Company B’s new employees were above Level 3. Company C had 20.69% of employees at Level 1, 62.07% at Level 2, and 17.24% at Level 3, while no employees had reached Level 4 by March 2021. Companies A and B demonstrated similarities in the transition between developmental levels among Level 3 and Level 4 new recruits, which occurred in the last month of the first year of hire. At the same time, the number of employees at Levels 2 and 3 had increased. However, the number of employees at all levels remained relatively stable in Company C. In Company C, the number of Level 3 employees decreased slightly in the last month. In Company B, the number of Level 1 employees decreased and those at Levels 2, 3, and 4 increased at the end of the fiscal year, as indicated in Fig. 5.

## Discussion and future implications

The two research questions were as follows: (1) How do new employees develop their skills, knowledge, and attitude in a company? and (2) What criteria can be used to define the development levels? For the first question, the skills, knowledge, and attitudes necessary to be acquired for the advancement of job tasks and a five-level model of development for new graduate employees were developed and proposed. The model was developed based on the Japanese context, in which various types of companies hire fresh graduate employees at the beginning of a fiscal year and provide training and support for this cohort of new recruits. The model’s development process involved detailed and rigorous validations steps referring to the IBSTPI competency development model. The scope of the model covers new graduate employees’ apprenticeships.

### How does a new employee develop their skills?

As shown in Table 5, a new employee starts learning the steps to execute manualized tasks one by one under the supervision of their managers, colleagues, and/or trainers. At Level 1, they usually comprehend what to do and how to follow instructions, and they take time to complete the task. At Level 2, they start to become aware of deadlines for their tasks, but they do not establish the necessary metacognition about their skills and knowledge; thus, they often take more time and do not complete the task in time. At Level 3, their apprenticeship reaches the intermediate level, and they start carrying out routine work without any problem with appropriate metacognition concerning work estimation. At Level 4, the type and variety of work they carry out begins to widen, and they begin to show their willingness to adapt to new tasks, increase their work knowledge, and learn from their colleagues and seniors. They start to apply their skills and knowledge to patterned work, which is not a routine work but work with certain rules and regulations. Patterned work requires a scheme that the new employees once used to carry out. Then, at Level 5, they begin to work as team members in the patterned work. They start to understand the effects and causes of their work by considering other workers and the business context. Then, around this time, they reach the end of their apprenticeship.

With the advent of the term “onboarding” and the gradual gain in popularity of research on workplace learning among new employees, serious problems concerning poor employee retention have become obvious. As Klein et al. (2015) stated, the significance of considering the different needs of newcomers and their onboarding processes reflects the needs of future research. In this research, we have focused on fresh graduate recruits and developed a model that closely observes their needs, challenges, and difficulties. Our findings provide information regarding the developmental path for “blank slate” newcomers who have just graduated from university and are embarking on their careers.

Apprenticeship has a long history in workplace learning since 1800 (Premont 1990) and is used for new employees’ training. However, the degree of apprenticeship is difficult for supervisors and trainers to determine and manage for providing effective support to new employees (Rowe et al. 2017). As Moon (2018) outlined, the concept of the apprenticeship can be applied from newcomer to professional, but in this research, apprenticeship is viewed as necessary until a worker becomes independent and learns how to develop their own skills. Previous research outlines the levels of apprenticeship in terms of learning process, outcome, and operational perspectives, but it does not provide detailed descriptions of an individual’s incremental development in a workplace in the manner of their performance. As discussed in the literature review section, the onboarding phase in the apprenticeship concept model of Moon (2018) provides the actions needed to support newcomers as they develop socialization skills, but it does not describe what to consider when performing the action. The proposed model in this research enables us to form more concrete images about the target employee’s level and what is necessary for them to develop further.

### What are criteria to discriminate the development levels?

Related to the second research question, two criteria have been proposed in this research. One is for the planning phase and the other is for the implementation phase of work tasks. Both criteria are reflected in the proposed model. Each phase of the criteria consists of three dimensions that demonstrate the salient differences among the levels, so that the criteria can discriminate the level from other levels of the target employee.

Nevertheless, the proposed development model includes detailed descriptions about new employees’ growth as workers, but it is difficult to diagnose and determine their level of development. The criteria were developed to provide a tool for both supervisors and employees themselves to determine a newcomer’s developmental level through performance statements. The IBSTPI’s competency development model in Fig. 1 shows performance statements as a final product along with competencies, and it was utilized as a framework in this study.

Validation of the model by looking at new graduate employees from three companies indicates the use of the proposed model with criteria that could demonstrate transitions of different developmental levels. Company B’s new employees showed relatively good self-development; the company had a market culture, and the results were valuable. At the stage of selection of the new employees, Company B carefully assessed the potential and basic learning and social skills of applicants when conducting hiring, and this may have affected the preferable transitions to the achieved level. Company A had a clan culture; it communicated within a family atmosphere and provided new employees with good interactions with trainers and colleagues. Employees with good human skills may perform and learn better in such a company. Company C had a strict hierarchical culture and did not place as much emphasis on new employees’ training, but instead attempted to control them. The transitions of the achieved levels in Company C reflected such a culture, and they remained stable throughout the months examined in this study. This result implies that the self-development of employees is difficult in such a culture.

The proposed model can be helpful for HR experts, researchers, practitioners, and even employees for designing and implementing training, a conducive environment, and support for new graduate employees’ appropriate development. The model was proposed, along with the performance criteria in the planning and implementation phases. These criteria can be used not only to assess employees’ levels of development, by showing expected behaviors before they reach the next level of development, but they can also be used as a rubric for employees as they reflect on their experience and work.

### Limitations of the study

The limitations of this research include a lack of validation for a variety of job types and ecological generalizability. Regarding job types, it was agreed among the researcher and the professionals who participated in this research that the model could be helpful regardless of the job type, since the proposed model indicates the development of the basic skills and knowledge that should be necessary for most job types. However, in this research, the validation of the model was conducted across a variety of job types and compared only among three companies. In the future, the proposed model should be examined for accuracy and appropriateness for different job types, especially in engineering and scientific professions.

The ecological generalizability of the model should also be tested. The model was developed based on data collected in Japan, although well-established conceptual theories and models were referred to during the process of developing the proposed model. Validation of our model should be carried out in other cultures and countries.

### Practical implications

As discussed by Backer and Bish (2019), previous work experience affects individual careers, and the current literature on onboarding deals with newcomers as a homogeneous group and “blank slates.” In this research focusing on new graduate employees, the newcomers were categorized into a relatively homogeneous group and blank slate. We believe that it is very important to provide new graduate employees who are filled with hope and expectation for their first experience of work with smooth and efficient onboarding. They construct their working experience from scratch. The model proposed in this research would be a guide for employers, employees, and especially trainers in terms of what to do and where to go next for their new graduate employees’ development. Using the model and determining the current level of the individual should provide helpful information for trainers to plan feedback and interactions with the new employee during OJT and to design training programs. Rauch et al. (2017) researched the effects of errors in the workplace and suggested that mistakes, along with in-depth analysis and proper reflection, may turn into learning opportunities. It is common for new employees to make mistakes and turn in inappropriate performance, but to provide them with good opportunities for learning, the model of the proper performances at each level may help new employees analyze their performance and mistakes in depth. The model and criteria proposed in this research could serve as a powerful tool for new employees to reflect on and self-evaluate their own work experiences and consider their next actions at their own pace for their own development. Goda et al. (2020) preliminary reported the design of a support system to improve interactions between new employees and their trainers and the self-reflections of new employees for their self-development, which would be among the possible applications of this model. The research findings could also be utilized to develop an effective support system for new employees. The proposed model and criteria can be utilized to facilitate the smooth onboarding of new colleagues in society.

## Conclusion

In this study, a five-level development model for new employees was proposed along with behavior criteria at different levels. The model was carefully developed referring the IBSTPI competency development model as a framework and based on data collected in Japan. New employees are simply graduates from high school or university and not exposed to organizational cultures and policies that may be unique in Japan. International cultures interact with each other (Bergiel et al. 2012) and mix together around the world. The results of this research may serve as a useful reference even in other countries besides Japan. The Japanese government set out five basic directions for future human resource development policies in 2020 (MHLW 2020): (a) the development of human resources for the realization of Society 5.0 and vocational training under “new daily life” after the COVID-19 pandemic, (b) support for the autonomous and independent career development of workers, (c) strengthening of the labor market infrastructure, (d) support for those who need special consideration, and (e) promotion of skills inheritance. This research is related to the second of these directions, and to provide effective support through both OJT and Off-JT, the steps and phases of new employees’ autonomous and independent career development should be identified. To reduce the early departure rate of the new employees, early adoption to a new environment and early success at the company are key, and the developmental stages and levels provided in this research will be useful for both designing and implementing training and mentoring, including for OJT and Off-JT. This research also provides criteria to comprehend the developmental level of new employees based on their behavior. The criteria could be used for new employees’ performance evaluation by their supervisors and also utilized to self-check and take guidance for developing necessary skills, knowledge, and attitudes to the next level.

## Data Availability

Due to the nature of this research, which was conducted under mutual agreements between the companies involved, the companies did not agree for their data to be shared publicly, so supporting data are not available.
